# Reducing oral frailty improves functional independence in older Brazilians: a causal inference study of disability-free survival using longitudinal modified treatment policies

**DOI:** 10.1093/geroni/igag091

**Published:** 2026-08-21

**Authors:** John Rong Hao Tay, Huihua Li, Karen G Peres, Rahul Malhotra, Anna Quialheiro, Eleonora D’Orsi, Marco A Peres

**Affiliations:** Health Services Research & Population Health Programme, Duke-NUS Medical School, Singapore, Singapore; National Dental Research Institute Singapore, National Dental Centre Singapore, Singapore, Singapore; National Dental Research Institute Singapore, National Dental Centre Singapore, Singapore, Singapore; Oral Health Academic Clinical Programme, Duke-NUS Medical School, Singapore, Singapore; National Dental Research Institute Singapore, National Dental Centre Singapore, Singapore, Singapore; Health Services Research & Population Health Programme, Duke-NUS Medical School, Singapore, Singapore; Centre for Ageing Research & Education, Duke-NUS Medical School, Singapore, Singapore; IA&Saúde - The Artificial Intelligence and Health Research Unit, Polytechnic University of Health, CESPU, Vila Nova de Famalicão, Portugal, Lisbon, Portugal; Department of Public Health, Federal University of Santa Catarina, Florianópolis, Brazil; Health Services Research & Population Health Programme, Duke-NUS Medical School, Singapore, Singapore; National Dental Research Institute Singapore, National Dental Centre Singapore, Singapore, Singapore

**Keywords:** Epidemiology, Oral health, Mastication, Public health, Health surveys

## Abstract

**Background and Objectives:**

Poor oral health is associated with functional decline and mortality in older adults, yet causal evidence linking oral health improvement with functional independence remains limited. We simulated the effects of hypothetical reductions in oral frailty on the cumulative probability of being alive and independent in basic activities of daily living (ADLs) among older adults in Brazil.

**Research Design and Methods:**

Data from the EpiFloripa Aging Cohort Study of community-dwelling adults aged 60 years or older in Florianópolis, Brazil, followed from 2009 to 2023, were analyzed (1702 participants). Oral frailty was measured at waves 1-3 using a seven-item composite score (range 0-7). The primary outcome was the cumulative probability of being alive and ADL-independent through wave 5, estimated using a discrete-time survival model. Longitudinal modified treatment policies with targeted maximum-likelihood estimation simulated nine oral health improvement scenarios, accounting for time-varying confounding and censoring.

**Results:**

336 deaths occurred during follow-up. A stepwise reduction in oral frailty increased the probability of being alive and ADL independent by 73% (probability ratio 1.73, 95% CI 1.48-2.02), corresponding to 15 829 additional functionally independent older adults per 100 000 individuals. Preventing edentulism with preserved masticatory function also improved the probability of being alive and ADL independent (probability ratio 1.21, 95% CI 1.08-1.36). Effects on survival alone were smaller and less certain.

**Discussion and Implications:**

Reducing oral frailty and preserving masticatory function increases functional independence among older adults, with smaller and uncertain effects on survival. Oral health is a modifiable determinant of healthy ageing.

Innovation and Translational Significance: Oral health is a neglected determinant of healthy aging. This study introduces a novel application of causal inference methods, longitudinal modified treatment policies, to a 14-year population-based cohort of Brazilian older adults, simulating nine hypothetical oral health improvement scenarios. We demonstrate that reducing oral frailty and preserving masticatory function could substantially increase the proportion of older adults remaining alive and functionally independent. These findings provide decision-makers with quantified, scenario-specific estimates of population gain, directly informing the integration of oral health into healthy aging policies and primary care strategies in middle-income countries.

## Background and objectives

Population aging is reshaping health systems worldwide.^1^ The central focus has shifted from survival alone to the preservation of functional independence. Extending life without maintaining functional independence, i.e., the ability to independently perform basic activities of daily living (ADLs), increases years lived with disability, long-term care needs, and healthcare costs.^2^ Remaining alive and independent in basic ADLs provides a policy-relevant indicator of healthy aging that integrates both longevity and quality of life.^3^

Oral health is increasingly recognized as an important determinant of healthy aging. Tooth loss and impaired chewing ability may compromise nutritional intake, promote systemic inflammation, and adversely affect psychosocial well-being, all of which contribute to declines in overall health and increased mortality.^4–8^ Despite this, oral conditions remain poorly integrated into primary care, chronic disease management, and healthy aging strategies, reflecting their historical marginalization from mainstream health systems.^9^ Consequently, their potential role as determinants of healthy aging, and their potential to extend years of independent living, have received limited attention in health policy and aging research. Because oral conditions are rarely fatal and dental care has been organized separately from the rest of medicine, oral diseases remain largely absent from global non-communicable disease and universal health coverage agendas, limiting recognition of oral health’s role in sustaining nutrition, physical function, and independent living in later life.^9–11^

The concept of oral frailty has been proposed to capture the cumulative burden of oral deficits that collectively impair daily functioning in older adults.^12^ Oral frailty refers to the age-related accumulation of oral health deficits, including tooth loss, impaired mastication, and dry mouth, that collectively compromise eating and daily function, and parallel broader physical decline (see Supplementary Material for detailed operationalization of the oral frailty measure used in this study). Unlike single-indicator approaches such as tooth loss alone, oral frailty reflects the multidimensional nature of oral health decline.^13^ Achieving improvements in oral health at scale could provide a promising strategy to support functional independence and survival. However, oral frailty and its determinants change over time, with confounders that may themselves be affected by earlier oral frailty—a setting in which conventional regression relating baseline exposures to later outcomes is prone to bias.^12^ As a result, prior studies have not quantified the population gains in functional independence that would follow from hypothetical, policy-relevant reductions in oral frailty over time.

To address these gaps, this study hypothesized that maintaining or improving oral health reduces oral frailty and increases the cumulative probability of remaining alive and independent in basic ADLs over time. Using data from a 14-year population-based cohort of Brazilian adults followed across four waves (2009-2024), we estimated the causal effects of reductions in oral frailty measured repeatedly in the first three waves, on the cumulative probability of being alive and independent in basic ADLs through the last wave, by simulating improvements in oral health trajectories and applying longitudinal modified treatment policies (LMTPs), which estimate these effects while accounting for time-varying exposures and confounders.

## Research design and methods

### Data and analytic sample

This study used data from the EpiFloripa Aging Cohort Study, a population-based prospective longitudinal study of community-dwelling adults aged 60 years and older in Florianópolis, Santa Catarina, Brazil. Participants were selected using a two-stage cluster sampling design. Detailed information on sampling and study procedures has been published elsewhere.^14^ The baseline survey (wave 1) was conducted in 2009-2010 using household interviews, with follow-up in-person interviews conducted in 2013-2014 (wave 2), 2017-2019 (wave 3), and 2023-2024 (wave 5). Wave 4 (2021-2022) was excluded from the present analysis because data collection was conducted exclusively by telephone during the COVID-19 pandemic, which did not include ascertainment of oral health exposure. Participants who completed a face-to-face interview at baseline were followed up. Although the EpiFloripa Aging study incorporated an open-cohort replenishment at wave 3, the present analysis was restricted to participants with a valid baseline interview at wave 1. Individuals missing a given follow-up wave were treated as censored thereafter.

Mortality information was ascertained at each follow-up wave through linkage with the Mortality Information System (SIM) of the Ministry of Health, from the Santa Catarina State Health Department, and via next-of-kin reports. Written informed consent was obtained from all participants. This manuscript follows the STROBE reporting guidelines.

### Exposure: oral frailty

The exposure was self-reported oral frailty, assessed at waves 1-3 using a score based on seven oral health conditions. (1) Complete edentulism was defined as the absence of all natural teeth in both the upper and lower arches (yes/no). (2) Self-rated oral health was assessed using the question “How would you rate your oral health?” and dichotomized as poor (very bad, bad, or fair) versus good (good or very good).^15^ (3) Perceived need for dental treatment was based on the question “Do you think you need dental treatment?” (yes/no). (4) Current use of complete dentures was assessed by asking “Do you wear a denture (full denture, total prosthesis)?” (yes/no). (5) Perceived need for complete dentures was defined as reporting a need for upper, lower, or both complete dentures (yes/no). Both items were retained as independent components of the oral frailty score, as a participant wearing a denture may still require a replacement or an opposing arch prosthesis. (6) Frequent xerostomia was assessed using the question “How often do you feel your mouth is dry?” and dichotomized as frequent (always or often) versus not frequent (sometimes, rarely or never). (7) Chewing difficulty was assessed using the question “How often do you have difficulty eating because of problems with your teeth or dentures?” and dichotomized as present (sometimes, often, or always) versus absent (never or rarely).

Each component was coded as present (1) or absent (0) and summed to generate an oral frailty score ranging from 0 to 7. These components were summed to produce the Oral Frailty Score (OFS) (0-7), which was subsequently categorized into three levels for analysis: 0-1 (low frailty), 2-3 (moderate frailty), and 4-7 (high frailty).^13^ This composite was operationalized following the deficit accumulation framework, wherein the cumulative burden across structural, symptomatic, and functional domains captures overall oral health vulnerability more completely than any single indicator (Supplementary Material). Composite oral frailty measures have demonstrated longitudinal validity for incident physical disability and mortality in community-dwelling older adults.^7^^,^^16^ Although oral frailty data were also assessed at wave 5, these data were not considered as an exposure as wave 5 represented the final outcome wave (so no subsequent outcome exists for it to precede).

To examine how score composition influenced the classification of oral frailty and estimated population-level effects, a supplementary analysis was conducted using a reduced 5-item OFS excluding self-rated oral health and perceived need for dental treatment. This reduced score, ranging from 0 to 5, was categorized using a parallel threshold structure (0-1: low frailty; 2-3: moderate frailty; 4-5: high frailty) to maintain consistency with the primary exposure specification.

### Outcome: alive and independent in basic ADLs

The primary outcome was the cumulative probability of being alive and independent in basic ADLs through the end of follow-up. Functional status was assessed using the Brazilian Multidimensional Functional Assessment Questionnaire, adapted from the Older Americans Resources and Services (BOMFAQ/OARS) instrument.^17^ Basic ADL limitations were used rather than instrumental ADLs, as the hypothesized biological pathways linking oral health to functional decline, through nutritional compromise, systemic inflammation, and reduced physical performance, are more likely to manifest as limitations in basic physical self-care than in complex socially mediated activities. At each follow-up wave, participants were classified as either alive and independent in basic ADLs (no limitations) or otherwise (dead or limitation in one or more basic ADLs). As a secondary outcome, survival alone (without considering ADL status) was examined across the same follow-up period.

### Covariates and censoring

Baseline confounders included age (years), sex, and ethnicity (self-reported using the Brazilian Institute of Geography and Statistics (IBGE) classification and dichotomized as white vs black, brown, and other, as the large majority of participants identified as white, with the remaining categories each representing small proportions of the sample. These variables were treated as time-invariant. Time-varying confounders, updated at each follow-up wave, included years of education, per-capita household income expressed in categories of Brazilian minimum wage (≤1, 1.1-5, 5.1-10, >10),^18^ household size (number of co-residents), self-rated general health (poor: very poor/poor/fair, good: good/very good),^19^ smoking status (never vs ever [former or current]), diabetes mellitus (yes/no), and cardiovascular disease (yes/no). Loss to follow-up due to nonresponse or being uncontactable was treated as censoring and accounted for in the analytic framework.

### Statistical analyses

**Figure 1:**
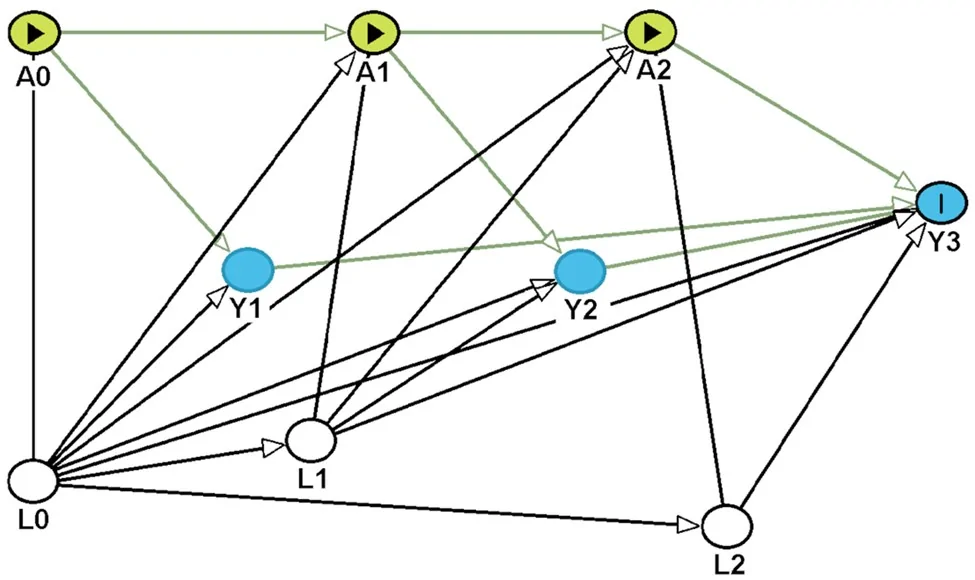
Proposed directed acyclic graph illustrating temporal associations among variables. L0 (baseline time-invariant covariates): age, sex, ethnicity, education. L0, L1, L2 (baseline time-varying covariates): per capita income, living arrangement, self-perceived health, smoking status, diabetes mellitus, cardiovascular disease. A0, A1, A2 (exposures): self-reported oral frailty at wave 1 (2009/2010), wave 2 (2013/2014), and wave 3 (2017/2019). Y1, Y2, Y3 (outcome): functional status and survival. The composite exposure and outcome are each represented as single nodes per wave; the covariates shown were selected as common causes shared across the domains of oral frailty and functional status and survival.

A series of LMTPs representing hypothetical modifications to oral health trajectories over follow-up were emulated (Figure 1). LMTPs estimate the mean counterfactual outcome under policies that shift the observed exposure according to a specified rule while maintaining the individual’s covariate and outcome history. This approach estimates the cumulative probability of being alive and independent in basic ADLs, or of survival alone, if oral health was improved under specific scenarios (for example, shifting individuals from higher to lower oral frailty categories), compared with the observed course. The LMTP framework accommodates time-varying exposures and confounders, accounts for informative censoring, and reduces the risk of positivity violations by avoiding unrealistic exposure assignments.^20^^,^^21^ Causal identification under this framework relies on the assumptions of no unmeasured confounding, positivity, and consistency. While exchangeability and consistency cannot be empirically verified, the positivity assumption can be evaluated using the observed data. This was done by examining the distribution of estimated treatment probabilities under each policy among individuals whose exposure would change, summarizing minimum estimated probabilities and the proportion of observations with very low probabilities (<1% and <5%) (Supplementary Table 1).

**Figure 2:**
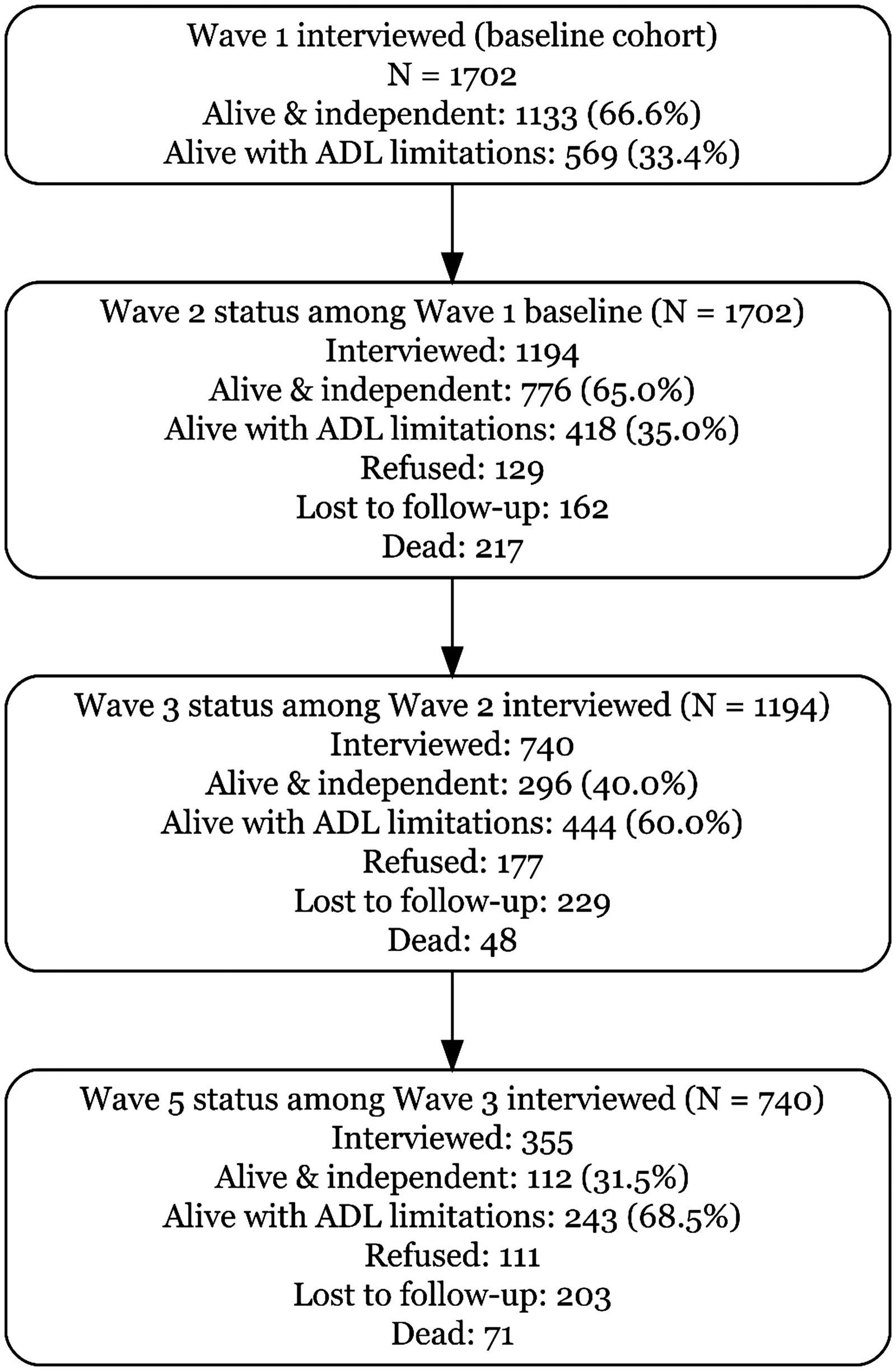
Flowchart of participants.

The primary outcome was analyzed using a discrete-time approach with three intervals corresponding to the follow-up waves (wave 1 to 2, wave 2 to 3, and wave 3 to 5). For each interval, the hazard of having at least one ADL limitation or being dead was estimated using covariates measured at wave 1. For the interval from wave 2 to 3, covariates measured at waves 1 and 2 were used. For the interval from wave 3 to 5, covariates measured at all three preceding waves were used. Participants with ADL limitations at baseline were retained in the analysis; their baseline ADL status was included as a covariate rather than used as an exclusion criterion. From wave 2 onwards, once a participant was ADL-dependent or dead, they were not included in subsequent intervals. Since only participants who were alive and ADL independent at the preceding wave remained in the risk set from wave 2 onwards, ADL status as a time-varying covariate was only informative at baseline. Baseline ADL status was therefore included as a covariate in all three interval models.

The cumulative probability of being alive and independent in basic ADLs through wave 5 was then obtained by multiplying the wave-specific survival probabilities across all three intervals. All-cause mortality was analyzed using the same discrete-time approach, with death recorded as having occurred or not between consecutive survey waves, yielding cumulative mortality risk through the end of follow-up.

Hypothetical scenarios were applied to time-varying oral health exposures measured at waves 1-3 and evaluated against the observed data. The following hypothetical scenarios were emulated. These scenarios were selected to reflect the range of oral health improvements that oral health services could work toward, from broad reductions in overall oral frailty to prevention of specific conditions such as edentulism and xerostomia. The estimated effects represent the maximum potential gain assuming complete achievement of each scenario and are intended to provide comparative benchmarks and policy targets:

#### Oral frailty category scenarios

- Shift individuals with high oral frailty to moderate frailty (OFS: 4-7 → 2-3)
- Shift individuals with high frailty to moderate frailty, and moderate frailty to low frailty (4-7 → 2-3; 2-3 → 0-1)
- Shift all individuals with high or moderate frailty directly to low frailty (2-7 → 0-1)

#### Functional oral health scenarios

- Eliminate masticatory impairment among individuals
- Eliminate masticatory impairment among denture users while retaining dentures
- Eliminate masticatory impairment by restoring function without dentures

#### Symptom- and tooth-based prevention scenarios

- Prevent xerostomia symptoms among individuals reporting dry mouth
- Prevent complete edentulism by maintaining non-edentulous status
- Prevent complete edentulism with preservation of masticatory function

Causal parameters under each policy were estimated using targeted maximum likelihood estimation (TMLE) within the LMTP framework,^21^ as implemented in the *lmtp* R package. Outcome, exposure, and censoring mechanisms were estimated using a SuperLearner ensemble comprising generalized linear models and generalized additive models, with 5-fold cross-validation. Exposure models used baseline and time-varying covariates; outcome and censoring models additionally included the concurrent exposure.

The primary causal contrasts were ratios and differences in the cumulative incidence of being alive and independent in basic ADLs through the end of follow-up—denoted as probability (risk) ratio and probability (risk) difference—comparing each policy with the observed data, with a value above 1 (above 0 for differences) indicating benefit. Absolute effects were expressed per 100,000 individuals—the “population gain”, defined as the additional number of older adults alive and independent under each policy. The same probability scale was used for the secondary overall-survival outcome.

Policy impact fractions (PIFs) express the proportional reduction in the adverse outcome expected under each policy relative to the observed data. For each policy, the PIF was calculated as the probability of the adverse outcome under the observed course, minus the probability under the policy, divided by the probability under the observed course;^22^ 95% confidence intervals were obtained by Monte Carlo simulation (10 000 draws) from the estimated probability ratios and their confidence limits. E-values were calculated for risk estimates, for both functional independence and overall survival outcomes.^23^ Missing data were handled using multiple imputation by chained equations with 20 imputed datasets. Variables with missing values were imputed using flexible machine-learning methods (random forest), while death indicators were not imputed (Supplementary Table 2).^24^ Final estimates were combined across imputations using Rubin’s rules. The extent of missing data across all variables and waves is summarized in Supplementary Table 1. Robustness to attrition was assessed by comparing baseline characteristics of participants retained, lost to follow-up, and deceased, by summarizing the distribution of estimated density ratios and the truncation applied (Figure S1), and by repeating the primary analysis as a complete-case analysis without imputation. A sensitivity analysis restricted to participants independent in basic ADLs at baseline was also conducted.

## Results

Of 1702 participants at baseline, 336 deaths were confirmed by the end of follow-up; among the 355 individuals interviewed at wave 5, 112 were alive and disability free, and 243 were alive with at least one basic ADL limitation (Figure 2). Baseline characteristics stratified by oral frailty category are presented in Table 1. Higher oral frailty was associated with older age, female sex, lower education, lower per-capita income, poorer self-rated health, and higher prevalence of diabetes and cardiovascular disease. Complete edentulism, denture use, xerostomia, and masticatory impairment were markedly more prevalent in the high oral frailty group. At baseline, 569 participants (33.4%) reported at least one basic ADL limitation. Participants retained at wave 5 and those lost to follow-up were broadly similar at baseline except for age (mean 67.1 vs 70.2 years), with comparable oral frailty and baseline ADL limitation (Supplementary Table 3).

**Table 1:** Survey-weighted baseline characteristics of the study sample overall and stratified by oral frailty category at baseline.

Variables	Overall *N *= 1702	Oral frailty category		
		Low (0-1) *n *= 529	Moderate (2-3) *n *= 750	High (4-7) *n *= 423
**Age, years**	70.6 (7.9)	69.3 (7.2)	71.0 (8.3)	71.6 (8.1)
**Sex**				
Male	37.5%	48.1%	33.5%	30.0%
Female	62.5%	51.9%	66.5%	70.0%
**Ethnicity^a^**				
White	86.5%	90.7%	85.7%	81.9%
Black, Brown, and other	13.5%	9.3%	14.3%	18.1%
**Education, years**	8.0 (5.8)	10.5 (6.2)	7.4 (5.4)	5.6 (4.5)
**Per-capita household income^b^ (BMW)**				
≤1	34.7%	27.2%	35.5%	43.9%
1.1-5	48.7%	48.2%	49.7%	47.4%
5.1-10	11.3%	15.1%	11.2%	6.0%
>10	5.3%	9.5%	3.6%	2.8%
**Household size (number of residents)**	3.2 (1.7)	3.1 (1.6)	3.2 (1.7)	3.2 (1.8)
**Self-rated health**				
Good	53.0%	67.5%	51.0%	35.7%
Poor	47.0%	32.5%	49.0%	64.3%
**Smoking status**				
Never	59.6%	64.0%	58.5%	55.5%
Ever (former or current)	40.4%	36.0%	41.5%	44.5%
Diabetes mellitus	21.5%	16.7%	22.2%	27.4%
Cardiovascular diseases	28.1%	22.6%	27.2%	37.9%
Any ADL limitation	32.5%	19.4%	32.6%	51.1%
Complete edentulism	32.3%	3.8%	43.3%	52.1%
**Self-rated oral health**				
Good	62.7%	93.7%	62.1%	19.0%
Poor	37.3%	6.3%	37.9%	81.0%
**Perceived need for dental treatment**	40.1%	17.8%	38.9%	74.9%
**Current use of complete dentures**	51.5%	11.5%	63.9%	85.4%
**Perceived need for full dentures**	37.8%	0.8%	38.8%	89.5%
**Frequent xerostomia**	16.8%	6.5%	16.0%	33.4%
**Masticatory impairment**	12.0%	0.5%	7.6%	37.4%

Abbreviations: ADL, activities of daily living. Values report percentage or mean (standard deviation).

a Ethnicity was self-reported using the IBGE classification. Black and Brown combines preto (*n *= 84) and pardo (*n *= 131); other includes amarelo (*n *= 12) and indígena (*n *= 16).

b Per-capita household income is expressed in Brazilian minimum wages (BMW), defined as total household income divided by the number of residents. During wave 1 (2009-2010), one Brazilian minimum wage corresponded to approximately R$488, equivalent to about USD 255-260.

Table 2 presents probability ratios for functional independence and survival under each hypothetical policy scenario. The largest gains were observed under scenarios targeting overall oral frailty. Shifting all individuals with moderate or high frailty directly to the lowest category (OFS 2-7 → 0-1) yielded a probability ratio of 1.71 (95% CI 1.46-2.02), corresponding to an estimated gain of 15,469 additional functionally independent older adults per 100 000 individuals (95% CI 11 072-19 867) and a PIF of 19.8% (95% CI 12.7-28.1%; Table 3). A stepwise downward shift (4-7 → 2-3; 2-3 → 0-1) produced similar results (probability ratio [PrR] 1.73, 95% CI 1.48-2.02), while shifting only high-frailty individuals to moderate frailty yielded a more modest but statistically significant improvement (PrR 1.20, 95% CI 1.08-1.34).

**Table 2:** Probability of disability-free survival across different exposures using simulated hypothetical scenarios.

Policy	PrR for functional independence and survival	95% CI	E-value
Shift individuals with high oral frailty to moderate (4-7 → 2-3)	1.20	1.08-1.34	1.69
Shift each category one level down (4-7 → 2-3; 2-3 → 0-1)	1.73	1.48-2.02	2.85
Shift all to low oral frailty (2-7 → 0-1)	1.71	1.46-2.02	2.82
Eliminate chewing difficulty among those affected	1.07	0.98-1.18	1.35
Eliminate chewing difficulty among denture users	1.07	1.00-1.14	1.34
Eliminate chewing difficulty by restoring function without dentures	1.09	1.02-1.17	1.41
Prevention of xerostomia symptoms among those affected	1.15	1.04-1.27	1.56
Prevention of complete edentulism (maintenance of non-edentulous status)	1.16	1.04-1.29	1.59
Prevention of complete edentulism with preservation of chewing function (maintenance of non-edentulous status)	1.21	1.08-1.36	1.72

Abbreviations: CI, confidence interval; PrR, probability ratio.

**Table 3:** Relative effects and policy impact fractions for disability-free survival under hypothetical oral-health scenarios.

Policy	Gain in functional independence and survival per 100,000 (95% CI)	PIF, % (95% CI)
Shift individuals with high oral frailty to moderate (4-7 → 2-3)	4339 (1485-7192)	5.51 (1.99-9.43)
Shift each category one level down (4-7 → 2-3; 2-3 → 0-1)	15 829 (11 698-19 959)	20.11 (13.50-28.34)
Shift all to low oral frailty (2-7 → 0-1)	15 469 (11,072-19 867)	19.82 (12.72-28.07)
Eliminate chewing difficulty among those affected	1581 (-533-3695)	2.03 (-0.59-4.83)
Eliminate chewing difficulty among denture users	1515 (106-2923)	1.93 (0.06-3.94)
Eliminate chewing difficulty by restoring function without dentures	2016 (611-3421)	2.58 (0.68-4.57)
Prevention of xerostomia symptoms among those affected	3181 (667-5696)	4.05 (1.03-7.28)
Prevention of complete edentulism (maintenance of non-edentulous status)	3440 (894-5985)	4.40 (1.16-8.09)
Prevention of complete edentulism with preservation of chewing function (maintenance of non-edentulous status)	4606 (1642-7570)	5.88 (2.21-9.99)

Abbreviations: CI, confidence interval; PIF, policy impact fraction.

The largest effects among component-specific scenarios included preventing complete edentulism with preserved masticatory function (PrR 1.21, 95% CI 1.08-1.36; gain of 4606 per 100 000; PIF 5.9%), and prevention of xerostomia (PrR 1.15, 95% CI 1.04-1.27; gain of 3181 per 100 000; PIF 4.1%). Scenarios targeting masticatory impairment alone produced smaller improvements, with probability ratios ranging from 1.07 to 1.09.

For overall survival, the estimated benefits were smaller and less certain (Supplementary Tables 4 and 5). Prevention of complete edentulism with preserved masticatory function showed the most survival benefit among component-specific scenarios (PrR 1.06, 95% CI 1.01-1.10), with an estimated 3595 fewer deaths per 100 000 individuals (95% CI 764-6427). Scenarios targeting overall oral frailty pointed in the same direction but with wider uncertainty for the larger shifts.

E-values ranged from 1.34 to 2.82, indicating that an unmeasured confounder would need to be associated with both the exposure and the outcome by probability ratios of at least this magnitude to fully explain away the observed effects. A supplementary analysis using the 5-item oral frailty score identified a substantially smaller high-frailty subgroup than the primary 7-item score (*n* = 101 vs *n* = 414), reflecting the high prevalence of self-rated oral health and perceived treatment need in this population. Estimated effects were in the same direction but attenuated in magnitude (PrR 1.07-1.15 for the three frailty category shift scenarios), consistent with the narrower population targeted under this scoring system (Supplementary Table 6). Positivity diagnostics confirmed adequate support for all hypothetical scenarios across the study population, with no shifted individuals having a scenario-assigned probability below 1% and fewer than 1% falling below 5% across all scenarios; effective sample size proportions ranged from 0.67 to 0.99 (Supplementary Table 1). In a sensitivity analysis restricted to participants independent in basic ADLs at baseline (*n* = 1133), the probability of remaining alive and independent increased under the oral-frailty-reduction scenarios, consistent with the primary analysis, although attenuated (Supplementary Table 7). In a complete-case analysis without multiple imputation, estimates were consistent with the primary analysis in direction and magnitude (Supplementary Table 8).

## Discussion and implications

### Main findings

This study provides evidence that oral health is a meaningful and potentially modifiable determinant of functional independence in later life. In this population-based cohort of Brazilian older adults followed over 14 years, improvements in oral health, particularly stepwise reductions in overall oral frailty and the prevention of tooth loss with preserved masticatory function, were associated with substantially higher probabilities of remaining alive and independent in ADLs. These estimates represent the gain under complete achievement of each scenario—the maximum that scenario would yield—and would be proportionally smaller under partial implementation. Simulated downward shifts in oral frailty yielded the largest estimated gains. A stepwise shift corresponded to a 20% increase in the probability of being alive and independent in ADLs, equivalent to an estimated 15 829 additional older adults independent in ADLs per 100 000 population. The comparable estimates for the stepwise shift and the direct shift to low frailty suggest a ceiling effect, whereby the marginal benefit of moving from moderate to low oral frailty appears small once high frailty has been eliminated. Effects on overall survival were smaller and less certain, suggesting that the primary benefit of better oral health lies not in extending life expectancy alone but in preserving functional capacity and delaying ADL limitations. These findings position oral health as an actionable target for policies aimed at increasing the proportion of older adults who remain alive and independent in ADLs. The findings were driven primarily by the preservation of functional independence rather than by reductions in mortality, and the policy estimates are best interpreted as idealized benchmarks and targets rather than directly achievable population interventions.

Our findings are consistent with prior evidence that poorer oral health is associated with increased risks of disability and death in older adults.^25^^,^^26^ Previous studies have generally reported modest-to-moderate associations between tooth loss, impaired chewing, or composite oral health measures and subsequent disability or mortality.^26–30^ Our results extend this evidence by estimating population-level gains expected under hypothetical oral health improvements, rather than comparing naturally occurring exposure groups. The primary outcome (i.e., remaining alive and independent in basic ADLs) is a composite endpoint that counts death as a failure (a participant who has died is no longer alive and independent), so the estimates reflect the maintenance of independent survival rather than longevity alone. Because functional independence and death are competing events that we did not separately model, we do not compare the magnitudes of their estimated effects. These findings for functional independence are nonetheless consistent with studies linking oral health to physical performance, frailty, and participation.^31–33^

The present study adopts a distinct methodological and conceptual approach to evaluating the relationship between oral health and aging outcomes. Most prior studies have relied on conventional regression or Cox proportional hazards models applied to baseline oral health exposures, which identify risk markers but do not address time-varying confounding or estimate the effects of improvements over time.^25^ Our study estimated the effects of hypothetical, policy-relevant changes in oral health trajectories rather than differences between naturally occurring exposure groups. By applying LMTPs combined with targeted maximum likelihood estimation, we explicitly simulated scenarios such as reducing oral frailty levels, preventing edentulism, or restoring chewing function, and then quantified the expected gains in functional independence and survival under those scenarios. This approach aligns more closely with questions faced by decision-makers—namely, how many additional older adults might remain independent if oral health services were strengthened—than with purely etiologic comparisons. Rather than asking what would happen if everyone were forced into an unrealistic exposure state, the analysis simulated targeted policy scenarios, such as moving from high to moderate or low oral frailty, which better reflects the scope of what oral health services may achieve at a population level.

Our operationalization of oral frailty as a cumulative deficit score reflects evidence that the accumulation of multiple oral health deficits is more strongly associated with adverse aging outcomes than any single indicator alone.^34^ Cumulative deficit approaches that incorporate perceived oral health burden have demonstrated longitudinal validity for incident frailty and adverse aging outcomes across multiple cohort studies,^34^^,^^35^ and our broader composite measure, incorporating perceived-need components, reflects the reality that in populations with pronounced inequalities in perceived oral health burden and unmet treatment need, these are clinically meaningful deficits in their own right.^35^^,^^36^ A supplementary analysis using the 5-item score, which excludes these subjective components, identified a substantially smaller high-frailty subgroup. Existing operationalizations of oral frailty vary considerably in their inclusion of subjective indicators,^37^ and the attenuation observed here reflects the narrower population targeted by the 5-item score rather than evidence against the primary findings.

### Strengths and limitations

Data were used from a large, population-based longitudinal cohort with more than a decade of follow-up, enabling examination of long-term functional outcomes. Second, the primary outcome focused on both survival and functional independence simultaneously, reflecting a clinically and policy-relevant indicator of healthy aging. We applied modern causal-inference methods that account for time-varying exposures and confounders, enabling estimation of effects under hypothetical scenarios that go beyond conventional aetiologic comparisons. The resulting estimates quantify expected population gains in independent living, providing a basis for translating oral health improvements that are directly interpretable for decision-makers. Finally, conducting this study in a population-based sample of Brazilian older adults characterized by wide socioeconomic inequalities and high burden of oral disease offers insights that are particularly relevant to middle-income countries seeking scalable strategies to promote functional independence in aging populations.

Oral health components and functional outcomes were largely self-reported and may be subject to measurement error or misclassification. As in all observational studies, residual confounding cannot be excluded despite the use of rich covariate data and causal methods designed to address time-varying confounding. E-values varied across scenarios, indicating that an unmeasured confounder would need to be associated with both oral frailty and the outcome by a probability ratio of at least the magnitude of the E-value to fully explain away the observed effects. Scenarios targeting overall oral frailty yielded higher E-values, suggesting these estimates are more robust to potential unmeasured confounding, while the smaller component-specific effects, particularly those targeting masticatory impairment alone, may be more susceptible to residual confounding. Moreover, because confounders were chosen as common causes shared across the oral-frailty domains, confounders specific to individual components (e.g., medications that induce xerostomia) may not be fully captured, which is most relevant to the component-specific scenarios.

Attrition over the 14-year follow-up was substantial. Although the analytic framework accounts for informative censoring, participants missing a given follow-up wave were not included in subsequent waves, and differential loss to follow-up among individuals with worse oral health or steeper functional decline may limit the generalizability of the findings. Multiple imputation and the censoring model address data missing at random conditional on measured covariates; residual departures from this assumption (e.g., loss to follow-up related to unmeasured factors) cannot be ruled out, although baseline characteristics were broadly similar between participants retained and lost to follow-up except for age (Supplementary Table 3). Further, the study was conducted in a single Brazilian urban cohort, and while the biological and functional mechanisms are likely generalizable, the effect sizes may differ across health systems and populations with different patterns of dental care access. The findings should therefore be interpreted as estimates of potential population gains under realistic improvements rather than definitive causal effects of specific clinical interventions. Stratified analyses by socioeconomic position were not feasible given subgroup sample sizes after attrition. Differential effects across socioeconomic groups represent an important direction for future research, particularly given Brazil’s marked income and education inequalities.

### Policy implications

Our findings suggest that oral health represents an underutilized lever for promoting functional independence in older adults. Under the simulated improvement scenarios, the proportion of older adults remaining alive and independent in ADLs could increase substantially at a population level. Brazil provides a particularly instructive context. Population aging has coincided with a high burden of oral disease and persistent socioeconomic inequalities.^38^ At the same time, Brazil has invested significantly in expanding oral health access through the Unified Health System (SUS), including the National Oral Health Policy (“Brasil Sorridente”), expanding primary care dental coverage nationwide. Despite this infrastructure, significant inequalities in oral health outcomes persist, with edentulism and unmet prosthetic need remaining disproportionately concentrated among older adults of lower socioeconomic position.^39^

Our findings suggest that strengthening and sustaining these investments, particularly in prosthetic rehabilitation, restorative care, and prevention of edentulism, could translate into meaningful gains in functional independence among older adults.^40^^,^^41^ Specific opportunities include embedding oral function assessment within routine geriatric evaluation in primary care, expanding access to full dentures and masticatory rehabilitation through SUS, and integrating oral health more explicitly within Brazil’s long-term care and healthy aging frameworks. Because these approaches rely largely on established, clinically routine interventions that can be delivered at scale, they represent opportunities to improve both quality of life and the number of years lived independently, while potentially reducing the societal and healthcare costs associated with disability and long-term care dependence. The cost-effectiveness of implementing these oral health improvements at scale warrants formal economic evaluation to inform resource allocation and health system planning.^42^^,^^43^

## Conclusions

Improving oral health, particularly by reducing oral frailty and preserving masticatory function and natural dentition, may substantially increase the probability of remaining alive and independent in basic ADLs among older adults, although effects on survival alone were smaller. These findings highlight oral health as a modifiable determinant of functional independence in later life, with implications for healthy aging policies and long-term care strategies.

## Supplementary Material

igag091_Supplementary_Data

## Data Availability

The data used in this study are from the EpiFloripa Aging Cohort Study and are not publicly available due to ethical and legal restrictions. De-identified data may be made available upon reasonable request to the EpiFloripa Aging Cohort Study research team and with approval from the Ethics Committee of the Federal University of Santa Catarina. This study was not preregistered.
