# Supplementary material for: Reducing oral frailty improves functional independence in older Brazilians: a causal inference study of disability-free survival using longitudinal modified treatment policies
Source: Innov Aging. 2026 Aug 21;10(10):igag091. doi: 10.1093/geroni/igag091 (PMC13616044; doi:10.1093/geroni/igag091)
Supplement: Supplementary file 1 [file igag091_supplementary_data.pdf]

***Innovation in Aging* Supplementary Material: Tay et al. Reducing oral frailty improves functional independence in older Brazilians: A causal inference study of disability-free survival using longitudinal modified treatment policies.**

Contents

Supplementary Text. Operationalisation of oral frailty 2

Supplementary Table 1. Positivity diagnostics for hypothetical oral frailty scenarios 4

Supplementary Table 2. Missing data summary 5

Supplementary Table 3. Baseline characteristics of participants retained at wave 5, lost to follow-up, and deceased 6

Supplementary Table 4. Probability of overall survival across different exposures using simulated hypothetical scenarios 6

Supplementary Table 5. Relative effects and policy impact fractions for overall survival under hypothetical oral-health scenarios. 7

Supplementary Table 6. Sensitivity analysis using a reduced 5-item oral frailty score: Probability of functional independence and overall survival across different exposures using simulated hypothetical scenarios 7

Supplementary Table 7. Sensitivity analysis restricted to participants independent in basic ADLs at baseline (n=1,133) 8

Supplementary Table 8. Complete-case sensitivity analysis (n=1,600) 8

Supplementary Figure 1. Distribution of estimated density-ratio weights (treatment policy and censoring) by the hypothetical scenarios, pooled over 20 imputations. 9

Censored observations receive weight 0 and are excluded; under the observed policy the ratio is 1. No weight exceeded 2.7. Density ratios were truncated at the 0.95 quantile and the clever covariate bounded at 30 9

STROBE Statement—Checklist of items that should be included in reports of *cohort studies* 10

Supplementary Text. Operationalisation of oral frailty

Oral frailty refers to the age-related accumulation of oral deficits across structural, symptomatic, and functional domains. Conceptually, it parallels the deficit-accumulation model of frailty used in geriatric medicine, in which vulnerability reflects the burden of multiple co-occurring deficits rather than any single impairment.[1,2] Published operationalisations vary in their component items and in their reliance on clinical examination versus self-report, but three main approaches can be distinguished.

First, oral frailty has been operationalised as a clinically assessed phenotype. In the Kashiwa cohort, oral frailty was defined as poor status in three or more of six measures: number of natural teeth, masticatory function, articulatory oral motor skill, tongue pressure, and self-reported difficulty eating and swallowing.[3] A simplified 5-item checklist subsequently combined fewer teeth, difficulty chewing, difficulty swallowing, dry mouth, and low articulatory oral motor skill, with oral frailty defined as two or more deficits.[4] Both approaches require objective assessment of tooth count and oral-motor function alongside selected self-reported items. Second, fully self-reported screening instruments, such as the 8-item Oral Frailty Index (OFI-8), assess related domains including masticatory function and swallowing difficulty, denture use, dry mouth, oral-health behaviours, and reduced social participation, without clinical examination.[5] Third, cumulative-deficit approaches sum the number of oral health problems present and use this count as a composite exposure, directly paralleling the frailty-index framework.[1,6] Both clinically assessed and self-reported oral frailty measures have been associated with incident physical frailty, functional disability, and mortality in community-dwelling older adults across Japanese, United Kingdom, and United States cohorts.[3-7] Meta-analytic evidence similarly supports associations between multidimensional oral health status and disability and mortality.[8]

Oral health-related quality of life (OHRQoL) instruments assess a related but distinct construct: the perceived impact of oral conditions on comfort, function, and psychosocial well-being. This well-developed family of validated patient-reported measures includes the Geriatric Oral Health Assessment Index (GOHAI), the Oral Health Impact Profile (OHIP and its short forms), and the Oral Impacts on Daily Performances (OIDP) scale, all of which have been systematically and psychometrically appraised for use in adult and older populations.[9-11] Unlike oral frailty indices, these instruments measure subjective quality-of-life burden rather than the accumulation of oral deficits. An OHRQoL instrument therefore would not have aligned with the exposure of interest in the present study. Moreover, no OHRQoL instrument was collected in the EpiFloripa Aging cohort; the available oral health assessment comprised the self-reported items used to construct the oral frailty score.

Drawing on this literature and a previously applied oral frailty measure from the EpiFloripa Aging cohort,[12] we operationalised oral frailty as a 7-item cumulative-deficit score

combining self-reported structural, symptomatic, functional, prosthetic, and perception-based indicators available consistently across waves. The inclusion of perception-based items is consistent with self-reported screening tools such as OFI-8, and their specific contribution was examined in a sensitivity analysis using a 5-item score that excluded self-rated oral health and perceived need for treatment. This self-reported cumulative-deficit approach is feasible for population-based ageing cohorts, does not require repeated clinical dental examination, and aligns with the deficit-accumulation framework shown to predict disability and mortality.

References

1. Searle SD, Mitnitski A, Gahbauer EA, Gill TM, Rockwood K. A standard procedure for creating a frailty index. BMC Geriatr. Sep 30 2008;8:24. doi:10.1186/1471-2318-8-24
2. Mitnitski AB, Mogilner AJ, Rockwood K. Accumulation of deficits as a proxy measure of aging. ScientificWorldJournal. Aug 8 2001;1:323-36. doi:10.1100/tsw.2001.58
3. Tanaka T, Takahashi K, Hirano H, et al. Oral Frailty as a Risk Factor for Physical Frailty and Mortality in Community-Dwelling Elderly. J Gerontol A Biol Sci Med Sci. Nov 10 2018;73(12):1661-1667. doi:10.1093/gerona/glx225
4. Tanaka T, Hirano H, Ikebe K, et al. Oral frailty five-item checklist to predict adverse health outcomes in community-dwelling older adults: A Kashiwa cohort study. Geriatr Gerontol Int. Sep 2023;23(9):651-659. doi:10.1111/ggi.14634
5. Tanaka T, Hirano H, Ohara Y, Nishimoto M, Iijima K. Oral Frailty Index-8 in the risk assessment of new-onset oral frailty and functional disability among community-dwelling older adults. Arch Gerontol Geriatr. May-Jun 2021;94:104340. doi:10.1016/j.archger.2021.104340
6. Kimble R, Papacosta AO, Lennon LT, et al. The Relationship of Oral Health with Progression of Physical Frailty among Older Adults: A Longitudinal Study Composed of Two Cohorts of Older Adults from the United Kingdom and United States. J Am Med Dir Assoc. Apr 2023;24(4):468-474.e3. doi:10.1016/j.jamda.2022.11.022
7. Dibello V, Lobbezoo F, Lozupone M, et al. Oral frailty indicators to target major adverse health-related outcomes in older age: a systematic review. Geroscience. Apr 2023;45(2):663-706. doi:10.1007/s11357-022-00663-8
8. Ko MJ, Seo S, So JS, et al. Deteriorated oral health and function as risk factors for physical disability and mortality in community-dwelling older adults: a systematic review and meta-analysis. Eur Geriatr Med. Oct 6 2025;doi:10.1007/s41999-025-01319-4
9. de Andrade FB, Lebrão ML, Santos JL, da Cruz Teixeira DS, de Oliveira Duarte YA. Relationship between oral health-related quality of life, oral health, socioeconomic, and general health factors in elderly Brazilians. J Am Geriatr Soc. Sep 2012;60(9):1755-60. doi:10.1111/j.1532-5415.2012.04104.x
10. Jiang CM, Chau RCW, McGrath C, Su N, Lo ECM. Instruments for assessing oral health-related quality of life (OHRQoL) in older adults: A meta-research study with standardized evaluation. J Dent. Nov 2025;162:106065. doi:10.1016/j.jdent.2025.106065
11. Riva F, Seoane M, Reichenheim ME, Tsakos G, Celeste RK. Adult oral health-related quality of life instruments: A systematic review. Community Dent Oral Epidemiol. Oct 2022;50(5):333-338. doi:10.1111/cdoe.12689
12. Mello A, Pereira MC, Figueiredo DR, D'Orsi E, Peres MA, Peres KG. Predictive models of weakness among older adults: the contribution of oral health indicators. Braz Oral Res. 2025;39:e064. doi:10.1590/1807-3107bor-2025.vol39.064

Supplementary Table 1. Positivity diagnostics for hypothetical oral frailty scenarios

Hypothetical scenario	Shifted (*N*)	Lowest 1% probability	Lowest 5% probability	Borderline support (<1%)	Borderline support (<5%)	ESS proportion
Shift high frailty to moderate (4–7 → 2–3)	414	0.332	0.383	0.0	0.0	0.99
Shift each category one level down	1,178	0.078	0.109	0.0	0.3	0.67
Shift all to low frailty (any → 0–1)	1,178	0.061	0.090	0.0	0.5	0.73
Eliminate chewing difficulty among those affected	200	0.683	0.728	0.0	0.0	0.99
Eliminate chewing difficulty among denture users	131	0.254	0.351	0.0	0.0	0.92
Eliminate chewing difficulty by restoring function without dentures	131	0.075	0.086	0.0	0.0	0.75
Prevention of xerostomia symptoms among those affected	260	0.587	0.670	0.0	0.0	0.99
Prevention of complete edentulism	581	0.148	0.212	0.0	0.0	0.78
Prevention of complete edentulism with preservation of chewing function	581	0.114	0.175	0.0	0.0	0.75

Note. Probabilities reflect the likelihood of each hypothetical scenario being plausible given a person's observed characteristics, estimated among individuals whose exposure would change under each scenario. Higher probabilities and ESS proportions close to 1 indicate better support for the scenario in the study population.
Abbreviations: ESS, effective sample size; OFS, oral frailty score.

Supplementary Table 2. Missing data summary

Variable	Wave	Number interviewed	Number missing (%)
Age	Wave 1	1,702	1 (0.1%)
Sex	Wave 1	1,702	0 (0.0%)
Ethnicity	Wave 1	1,702	52 (3.1%)
Education	Wave 1	1,702	8 (0.5%)
Per-capita income	Wave 1	1,702	0 (0.0%)
Household size	Wave 1	1,702	0 (0.0%)
Self-rated health	Wave 1	1,702	50 (2.9%)
Smoking status	Wave 1	1,702	2 (0.1%)
Diabetes mellitus	Wave 1	1,702	0 (0.0%)
Cardiovascular disease	Wave 1	1,702	0 (0.0%)
Oral frailty score	Wave 1	1,702	92 (5.4%)
Education	Wave 2	1,195	3 (0.3%)
Per-capita income	Wave 2	1,195	50 (4.2%)
Household size	Wave 2	1,195	2 (0.2%)
Self-rated health	Wave 2	1,195	56 (4.7%)
Smoking	Wave 2	1,195	1 (0.1%)
Diabetes mellitus	Wave 2	1,195	0 (0.0%)
Cardiovascular disease	Wave 2	1,195	0 (0.0%)
Oral frailty score	Wave 2	1,195	57 (4.8%)
Education	Wave 3	742	0 (0.0%)
Per-capita income	Wave 3	742	1 (0.1%)
Household size	Wave 3	742	1 (0.1%)
Self-rated health	Wave 3	742	34 (4.6%)
Smoking status	Wave 3	742	0 (0.0%)
Diabetes mellitus	Wave 3	742	0 (0.0%)
Cardiovascular disease	Wave 3	742	0 (0.0%)
Oral frailty score	Wave 3	742	49 (6.6%)

Supplementary Table 3. Baseline characteristics of participants retained at wave 5, lost to follow-up, and deceased

Variable	Retained (W5)	Lost to follow-up	Died	SMD (retained vs lost)
N	355	1011	336	
Age, years	67.1 (5.8)	70.2 (7.3)	76.0 (9.3)	-0.46
Female, %	70.4	63.8	57.4	0.14
White, %	86.4	86.1	81.1	0.01
Education, years	8.3 (5.7)	7.9 (6.0)	6.1 (5.3)	0.08
Per-capita income ≤1 (BMW), %	37.7	34.6	40.8	0.07
Household size (number of residents)	3.2 (1.7)	3.2 (1.7)	3.4 (1.8)	0.01
Good self-rated health, %	56.5	54.3	34.6	0.04
Ever smoker, %	34.6	37.7	47.5	-0.06
Diabetes, %	17.2	20.9	30.7	-0.09
Cardiovascular disease, %	23.1	27.0	36.6	-0.09
Any ADL limitation, %	25.9	28.6	56.0	-0.06
Oral frailty score (0-7)	2.2 (1.5)	2.3 (1.6)	2.8 (1.5)	-0.05
High oral frailty (OFS 4-7), %	19.9	23.3	31.2	-0.08

Notes. Values are mean (SD) for continuous variables and % for binary; SMD, standardised difference (retained vs lost to follow-up).

Supplementary Table 4. Probability of overall survival across different exposures using simulated hypothetical scenarios

Policy	PrR for overall survival	95% CI	E-value
Shift individuals with high oral frailty to moderate (4–7 → 2–3)	1.04	0.99-1.09	1.25
Shift each category one level down (4–7 → 2–3; 2–3 → 0–1)	1.04	0.96-1.13	1.24
Shift all to low oral frailty (2–7 → 0–1)	1.06	0.94-1.18	1.30
Eliminate chewing difficulty among those affected	1.02	0.98-1.05	1.15
Eliminate chewing difficulty among denture users	1.01	0.99-1.04	1.13
Eliminate chewing difficulty by restoring function without dentures	1.02	0.99-1.04	1.14
Prevention of xerostomia symptoms among those affected	1.00	0.96-1.04	1.06
Prevention of complete edentulism (maintenance of non-edentulous status)	1.05	1.00-1.11	1.29
Prevention of complete edentulism with preservation of chewing function (maintenance of non-edentulous status)	1.06	1.01-1.10	1.30

Abbreviations: CI, confidence interval; PrR, probability ratio.

Supplementary Table 5. Relative effects and policy impact fractions for overall survival under hypothetical oral-health scenarios.

Policy	Gain in overall survival per 100,000 (95% CI)	PIF (95% CI)
Shift individuals with high oral frailty to moderate (4–7 → 2–3)	2,656 (-31-5,744)	3.90 (-0.74-8.32)
Shift each category one level down (4–7 → 2–3; 2–3 → 0–1)	2,518 (-3,068-8,104)	3.64 (-4.40-11.53)
Shift all to low oral frailty (2–7 → 0–1)	3,638 (-3,932-11,208)	5.40 (-5.94-15.43)
Eliminate chewing difficulty among those affected	1,063 (-1,028-3,154)	1.63 (-1.61-4.68)
Eliminate chewing difficulty among denture users	847 (-752-2,45)	1.28 (-1.23-3.75)
Eliminate chewing difficulty by restoring function without dentures	847 (-752-2,445)	1.54 (-0.92-3.91)
Prevention of xerostomia symptoms among those affected	-237 (-2,744-2,270)	-0.38 (-4.39-3.36)
Prevention of complete edentulism (maintenance of non-edentulous status)	3,466 (-78-6,854)	5.09 (0.25-9.79)
Prevention of complete edentulism with preservation of chewing function (maintenance of non-edentulous status)	3,595 (764-6,427)	5.25 (1.15-9.18)

Abbreviations: CI, confidence interval; PIF, policy impact fraction.

Supplementary Table 6. Sensitivity analysis using a reduced 5-item oral frailty score: Probability of functional independence and overall survival across different exposures using simulated hypothetical scenarios

Policy	PrR for overall survival	95% CI	E-value
**Functional independence**			
Shift individuals with high oral frailty to moderate (4–5 → 2–3)	1.07	1.02-1.13	1.34
Shift each category one level down (4–5 → 2–3; 2–3 → 0–1)	1.13	0.95-1.35	1.52
Shift all to low oral frailty (2–5 → 0–1)	1.15	0.97-1.37	1.56
**Overall survival**			
Shift individuals with high oral frailty to moderate (4–5 → 2–3)	1.01	0.98-1.03	1.09
Shift each category one level down (4–5 → 2–3; 2–3 → 0–1)	1.03	0.94-1.13	1.20
Shift all to low oral frailty (2–5 → 0–1)	1.03	0.94-1.13	1.21

Abbreviations: CI, confidence interval; PrR, probability ratio.

Supplementary Table 7. Sensitivity analysis restricted to participants independent in basic ADLs at baseline (n=1,133)

Scenario	PrR (95% CI)	Gain per 100,000 (95% CI)	PIF, % (95% CI)
Shift high oral frailty to moderate (OFS 4–7 → 2–3)	1.12 (0.99–1.26)	3,303 (-456 to 7,062)	4.6 (-0.5–10.3)
Stepwise shift (4–7 → 2–3; 2–3 → 0–1)	1.53 (1.29–1.82)	15,142 (8,356 to 21,929)	21.1 (11.8–32.9)
Shift all moderate/high oral frailty to low (2–7 → 0–1)	1.64 (1.34–2.01)	18,333 (9,823 to 26,842)	25.8 (13.5–40.7)
Eliminate masticatory impairment among individuals	1.06 (0.96–1.17)	1,699 (-1,193 to 4,590)	2.4 (-1.5–6.6)
Eliminate masticatory impairment among denture users (dentures retained)	0.99 (0.95–1.04)	-272 (-1,542 to 999)	-0.4 (-2.2–1.5)
Eliminate masticatory impairment by restoring function without dentures	1.01 (0.97–1.05)	287 (-879 to 1,453)	0.4 (-1.2–2.1)
Prevent xerostomia among those affected	1.14 (1.04–1.26)	4,070 (820 to 7,320)	5.7 (1.4–10.3)
Prevent complete edentulism (maintain non-edentulous status)	1.12 (0.99–1.26)	3,380 (-260 to 7,021)	4.7 (-0.2–10.4)
Prevent complete edentulism with preserved masticatory function	1.19 (1.06–1.34)	5,453 (1,539 to 9,366)	7.6 (2.3–13.7)

Abbreviations: PrR, probability ratio; PIF, policy impact fraction.

Supplementary Table 8. Complete-case sensitivity analysis (n=1,600)

Scenario	PrR (95% CI)	Gain per 100,000 (95% CI)	PIF, % (95% CI)
Shift high oral frailty to moderate (OFS 4–7 → 2–3)	1.21 (1.07–1.34)	4,645 (1,649 to 7,641)	6.0 (2.2–10.2)
Stepwise shift (4–7 → 2–3; 2–3 → 0–1)	1.69 (1.46–1.91)	15,447 (11,955 to 18,939)	19.9 (13.9–27.1)
Shift all moderate/high oral frailty to low (2–7 → 0–1)	1.68 (1.43–1.93)	15,369 (11,634 to 19,103)	19.9 (13.0–27.8)
Eliminate masticatory impairment among individuals	1.08 (0.99–1.17)	1,831 (-247 to 3,909)	2.4 (-0.3–5.2)
Eliminate masticatory impairment among denture users (dentures retained)	0.95 (0.91–1.00)	-1,019 (-2,017 to -21)	-1.3 (-2.6–0.1)
Eliminate masticatory impairment by restoring function without dentures	1.01 (0.98–1.05)	331 (-495 to 1,157)	0.4 (-0.6–1.5)
Prevent xerostomia among those affected	1.15 (1.03–1.26)	3,284 (707 to 5,860)	4.2 (1.1–7.6)
Prevent complete edentulism (maintain non-edentulous status)	1.18 (1.06–1.31)	4,073 (1,440 to 6,705)	5.3 (1.8–9.2)
Prevent complete edentulism with preserved masticatory function	1.20 (1.07–1.33)	4,523 (1,779 to 7,268)	5.8 (2.3–9.8)

Abbreviation: PrR, probability ratio.

Supplementary Figure 1. Distribution of estimated density-ratio weights (treatment policy and censoring) by the hypothetical scenarios, pooled over 20 imputations. Censored observations receive weight 0 and are excluded; under the observed policy the ratio is 1. No weight exceeded 2.7. Density ratios were truncated at the 0.95 quantile and the clever covariate bounded at 30

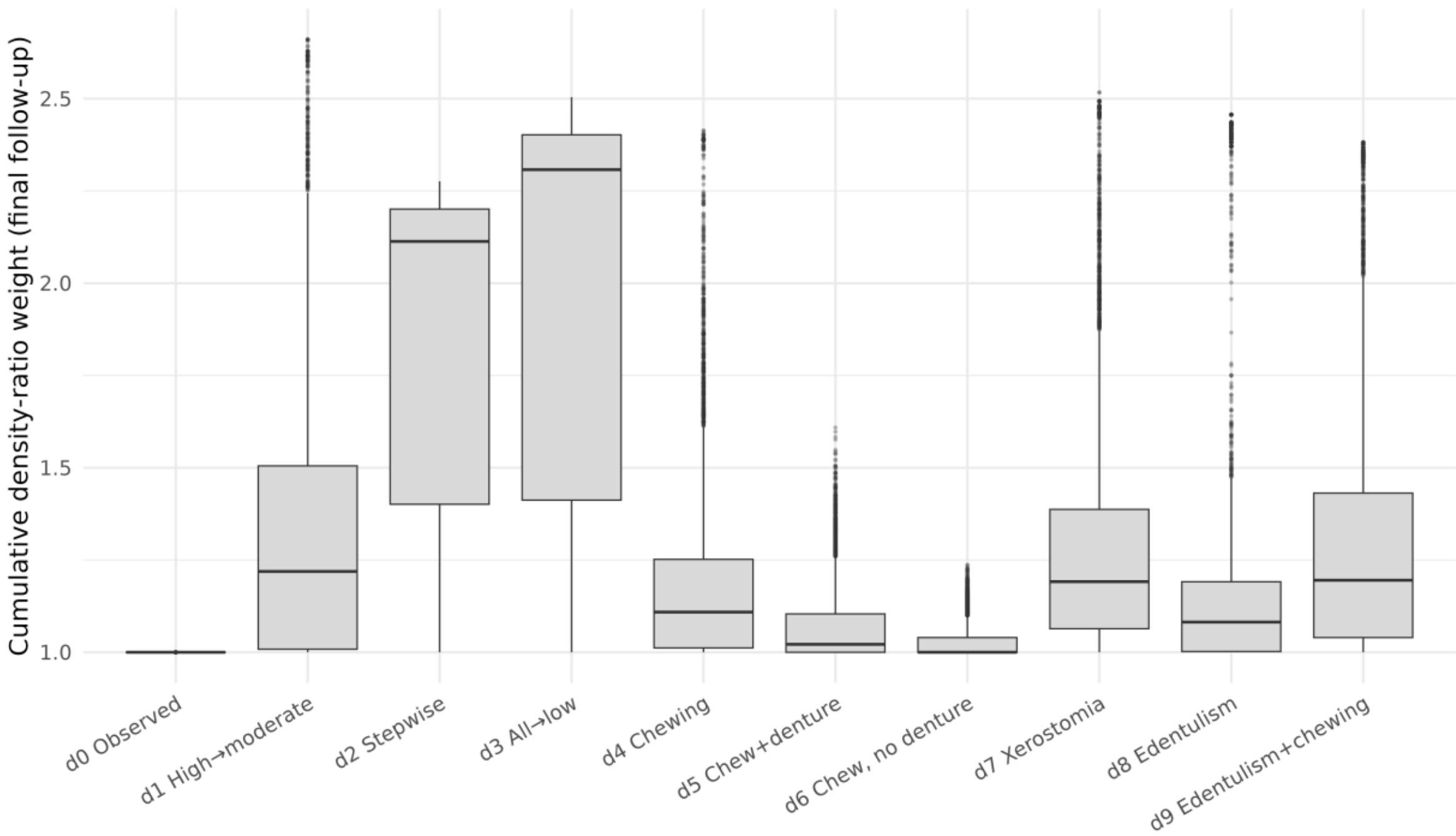

STROBE Statement—Checklist of items that should be included in reports of *cohort studies*

	Item No	Recommendation	Page No
**Title and abstract**	1	(*a*) Indicate the study's design with a commonly used term in the title or the abstract<br>(*b*) Provide in the abstract an informative and balanced summary of what was done and what was found	1
**Introduction**			
Background/rationale	2	Explain the scientific background and rationale for the investigation being reported	3
Objectives	3	State specific objectives, including any prespecified hypotheses	4
**Methods**			
Study design	4	Present key elements of study design early in the paper	4-5
Setting	5	Describe the setting, locations, and relevant dates, including periods of recruitment, exposure, follow-up, and data collection	4-5
Participants	6	(*a*) Give the eligibility criteria, and the sources and methods of selection of participants. Describe methods of follow-up<br>(*b*) For matched studies, give matching criteria and number of exposed and unexposed	4-5
Variables	7	Clearly define all outcomes, exposures, predictors, potential confounders, and effect modifiers. Give diagnostic criteria, if applicable	4-6
Data sources/ measurement	8*	For each variable of interest, give sources of data and details of methods of assessment (measurement). Describe comparability of assessment methods if there is more than one group	4-6
Bias	9	Describe any efforts to address potential sources of bias	4-6
Study size	10	Explain how the study size was arrived at	4-6
Quantitative variables	11	Explain how quantitative variables were handled in the analyses. If applicable, describe which groupings were chosen and why	4-6
Statistical methods	12	(*a*) Describe all statistical methods, including those used to control for confounding<br>(*b*) Describe any methods used to examine subgroups and interactions<br>(*c*) Explain how missing data were addressed<br>(*d*) If applicable, explain how loss to follow-up was addressed<br>(*e*) Describe any sensitivity analyses	4-7
**Results**			
Participants	13*	(a) Report numbers of individuals at each stage of study—eg numbers potentially eligible, examined for eligibility, confirmed eligible, included in the study, completing follow-up, and analysed<br>(b) Give reasons for non-participation at each stage	8, Figure 2

	Item No	Recommendation	Page No
		(c) Consider use of a flow diagram	
Descriptive data	14*	(a) Give characteristics of study participants (eg demographic, clinical, social) and information on exposures and potential confounders<br>(b) Indicate number of participants with missing data for each variable of interest<br>(c) Summarise follow-up time (eg, average and total amount)	8, Figure 2, Supplementary Table 2
Outcome data	15*	Report numbers of outcome events or summary measures over time	8, Table 2, Supplementary Table 3-4
Main results	16	(*a*) Give unadjusted estimates and, if applicable, confounder-adjusted estimates and their precision (eg, 95% confidence interval). Make clear which confounders were adjusted for and why they were included<br>(*b*) Report category boundaries when continuous variables were categorized<br>(*c*) If relevant, consider translating estimates of relative risk into absolute risk for a meaningful time period	8-9, Table 2, Supplementary Table 3-4
Other analyses	17	Report other analyses done—eg analyses of subgroups and interactions, and sensitivity analyses	8-9, Supplementary Table 1,5
**Discussion**			
Key results	18	Summarise key results with reference to study objectives	9-10
Limitations	19	Discuss limitations of the study, taking into account sources of potential bias or imprecision. Discuss both direction and magnitude of any potential bias	10-11
Interpretation	20	Give a cautious overall interpretation of results considering objectives, limitations, multiplicity of analyses, results from similar studies, and other relevant evidence	10-12
Generalisability	21	Discuss the generalisability (external validity) of the study results	10-12
**Other information**			
Funding	22	Give the source of funding and the role of the funders for the present study and, if applicable, for the original study on which the present article is based	Non-anonymised title page

*Give information separately for exposed and unexposed groups.

Note: An Explanation and Elaboration article discusses each checklist item and gives methodological background and published examples of transparent reporting. The STROBE checklist is best used in conjunction with this article (freely available on the Web sites of PLoS Medicine at http://www.plosmedicine.org/, Annals of Internal Medicine at http://www.annals.org/, and Epidemiology at http://www.epidem.com/). Information on the STROBE Initiative is available at http://www.strobe-statement.org.
